# Microsatellite Instability Status and Mismatch Repair Defects Testing in Endometrial Cancer—Insights from the Multicenter E-PEC Trial

**DOI:** 10.3390/diagnostics16010100

**Published:** 2025-12-27

**Authors:** Büsra Eser, David Papior, Jon Salmanton-García, Oliver A. Cornely, Bernd Morgenstern, Clarissa Herpel, Julia C. Radosa, Anas Almuheimid, Bahriye Aktas, Laura Weydandt, Julia Wittenborn, Philipp Meyer-Wilmes, Verena Friebe, Christiane Leidinger, Rainer Kimmig, Fabinshy Thangarajah

**Affiliations:** 1 Department of Gynecology and Obstetrics, Medical Faculty, University Hospital of Essen, University of Duisburg-Essen, 45147 Essen, Germanyfabinshy.thangarajah@uk-essen.de (F.T.); 2 Institute of Translational Research, Cologne Excellence Cluster on Cellular Stress Responses in Aging-Associated Diseases (CECAD), Faculty of Medicine, University Hospital Cologne, University of Cologne, 50931 Cologne, Germany; 3 Clinical Trials Centre Cologne (ZKS Köln), Faculty of Medicine, University Hospital Cologne, University of Cologne, 50931 Cologne, Germany; 4 Department I of Internal Medicine, Center for Integrated Oncology Aachen Bonn Cologne Duesseldorf (CIO ABCD), University of Cologne, 50931 Cologne, Germany; 5 Department I of Internal Medicine, European Confederation for Medical Mycology (ECMM) Excellence Center, University of Cologne, 50931 Cologne, Germany; 6 German Centre for Infection Research (DZIF), Partner Site Bonn-Cologne, 50931 Cologne, Germany; 7 Department of Gynecology and Gynecologic Oncology, Center for Integrated Oncology (CIO Aachen, Bonn, Cologne, Düsseldorf), Faculty of Medicine, University Hospital Cologne, University of Cologne, 50937 Cologne, Germany; 8 Department of Gynecology and Obstetrics, University Hospital of Homburg, 66424 Homburg, Germany; 9 Department of Gynecology, University Hospital of Leipzig, 04103 Leipzig, Germany; 10 Comprehensive Cancer Center Central Germany, Partner Site Leipzig, 04103 Leipzig, Germany; 11 Department of Obstetrics and Gynecology, Center for Integrated Oncology (CIO Aachen, Bonn, Cologne, Düsseldorf), University Hospital of RWTH Aachen, 52074 Aachen, Germany; 12 Department of Gynecology and Obstetrics, University Hospital of Düsseldorf, 40225 Düsseldorf, Germany

**Keywords:** endometrial cancer, microsatellite instability, mismatch repair-testing, molecular characterization, immune checkpoint inhibition

## Abstract

**Background:** Mismatch repair (MMR) and microsatellite instability (MSI) testing have become essential biomarkers in the molecular classification of endometrial cancer (EC), guiding adjuvant treatment decisions and eligibility for immune checkpoint inhibition. Although international guidelines recommend universal testing, real-world implementation remains heterogeneous. This study aimed to evaluate trends in MMR and MSI testing and associated molecular diagnostics in Germany between 2018 and 2022. **Methods:** A retrospective multicenter analysis was conducted across German tertiary care centers. Data from patients with histologically confirmed EC between 2018 and 2022 were extracted from standardized electronic pathology records. Annual testing rates for *MSI*, *MMR*, *POLE*, *TP53*, and L1CAM were analyzed using descriptive statistics and trend analysis (Chi-square test for trend, *p* < 0.05). Therapeutic data were collected to assess the use of immune checkpoint inhibitors. **Results:** There was a significant increase in the annual rates of molecular testing for MSI, *POLE*, *TP53*, and L1CAM over the five-year observation period (all *p* < 0.05). TP53 testing showed the highest increase (13.1% → 78.6%), while MSI testing rose from 82.9% to 97.4%. Both *POLE* and L1CAM testing were virtually absent in 2018 (0% and 1.6%) but reached 15.7% by 2022. **Conclusions:** This study demonstrates a rapid and substantial implementation of MMR and MSI testing in German clinical practice, reflecting successful translation of trial results into routine care. However, implementation of testing in guidelines appeared time-shifted. For bridging this gap, annual guideline updates seem to be necessary.

## 1. Introduction

Endometrial cancer (EC) is the most common gynecological malignancy among women and presents a significant challenge in oncology. In Germany, approximately 11,200 new cases were projected for the year 2020 [1]. Most patients are diagnosed at an early stage, which contributes to a favorable overall prognosis, with a five-year survival rate of about 78% [2]. However, specific molecular subtypes are associated with poorer outcomes, particularly tumors characterized by microsatellite instability (MSI) or mismatch repair (MMR) deficiency.

These biomarkers are increasingly important for risk stratification, therapeutic decision-making, and genetic counseling [3]. Tumors with MSI show favorable responses to immune checkpoint inhibitors and often benefit more from adjuvant radiotherapy, whereas gestagen-based regimens and platinum-based chemotherapy appear less effective in this subgroup [4,5].

Historically, MMR/MSI testing in endometrial cancer has only recently been implemented into routine clinical recommendations. In Germany, the first national endorsement was included in the S3 guideline (2018), which recommended MMR testing to improve Lynch syndrome detection [4]. In Germany, national S3 guidelines represent evidence-based, consensus-driven clinical practice guidelines developed under the coordination of the Association of the Scientific Medical Societies in Germany (AWMF). These guidelines define the standard of care in clinical practice by integrating the highest level of available evidence with structured multidisciplinary expert consensus and are regularly updated to guide diagnosis and treatment decisions [4].

This was followed by the ESGO/ESTRO/ESP guidelines (2020, published 2021), which for the first time advocated universal MMR or MSI testing across Europe [6]. Finally, the 2022 update of the German S3 guideline consolidated this recommendation by mandating routine testing in all newly diagnosed cases [7].

In this context, the implementation of molecular diagnostics represents a paradigm shift from purely histopathological risk assessment toward biologically informed treatment stratification. While international guidelines increasingly emphasize comprehensive molecular classification, national guideline adoption and real-world implementation often occur with a temporal delay.

The introduction of MMR testing into the German S3 guideline initially focused on hereditary cancer risk assessment; however, accumulating evidence has underscored its broader therapeutic relevance, particularly regarding immunotherapy eligibility and molecular risk stratification. Evaluating how rapidly and consistently these recommendations have been translated into routine clinical practice is therefore essential to assess the effectiveness of guideline implementation and to identify potential gaps between evidence, recommendations, and patient care.

Consequently, the years 2018 to 2022 represent a critical period during which MMR/MSI testing transitioned from a novel recommendation to an established standard of care. The GO WEST (Gynäkologische Onkologie West) study group conducted the E-PEC (“Early implementation of practice-changing study results and guidelines in the surgical treatment of patients with endometrial cancer”) study to analyze real-world data from patients undergoing primary surgery for endometrial cancer between 2018 and 2022 at six German university hospitals.

The primary aim of this study was to assess the implementation of MMR/MSI testing in German clinical practice and to identify barriers and opportunities for advancing personalized therapy in endometrial cancer.

## 2. Materials and Methods

This multicenter retrospective study included women with newly diagnosed endometrial carcinoma who underwent primary surgery between 2018 and 2022 at six academic hospitals in Germany.

Inclusion criteria were women with newly diagnosed, histologically confirmed endometrial carcinoma who underwent primary surgical treatment between 2018 and 2022 at one of the six participating German academic centers.

Exclusion criteria included patients who received primary chemoradiation or any systemic therapy prior to surgery.

Comprehensive data on patient demographics, histological characteristics, molecular markers, and treatment details were systematically recorded in the E-PEC database across all participating centers.

The E-PEC (Endometrial Cancer—Pathology, Epidemiology, and Care) database is a multicenter clinical and pathological registry designed to capture real-world data on endometrial cancer in German tertiary care centers. It includes standardized information on tumor histology, staging, molecular biomarkers, and administered therapies. Data entry follows predefined variables and harmonized definitions to ensure interinstitutional comparability, with quality control measures including regular plausibility checks and centralized data monitoring.

Molecular and pathological variables extracted for analysis included estrogen receptor (ER), progesterone receptor (PR), L1 cell adhesion molecule (L1CAM), lymphovascular space invasion (LVSI), *TP53* status, *POLE* alterations, and microsatellite instability (MSI) or mismatch repair (MMR) status. MMR and MSI were assessed according to institutional protocols using immunohistochemistry (IHC) for *MutL homolog 1* (*MLH1*), *MutS homolog 2* (*MSH2*), *MutS homolog 6* (*MSH6*), and *postmeiotic segregation increased 2 *(*PMS2*) and/or polymerase chain reaction (PCR)-based MSI testing.

### Statistical Analysis

Statistical analyses were performed using SPSS version 28.0 (IBM Corp., Armonk, NY, USA). Continuous variables were summarized as medians and interquartile ranges (IQR), as distributions were non-normal based on visual inspection of histograms. Categorical variables were reported as counts and percentages. Comparisons of categorical variables were conducted using the Chi-square test when all expected cell frequencies were ≥5; otherwise, Fisher’s exact test was applied. Continuous variables were compared using the Mann–Whitney U test. A *p*-value < 0.05 was considered statistically significant.

The study was conducted in accordance with the Declaration of Helsinki. Ethical approval was obtained from the Ethics Committee of the Medical Faculty of the University of Duisburg-Essen (Approval No. 24-11932-BO, issued on 2 July 2024). Patient consent was waived due to the retrospective design of the study.

## 3. Results

In total, 504 patients met the inclusion criteria. Patient characteristics are summarized in Table 1. The median age was 63 years (IQR = 14.75), with a median BMI of 30 kg/m^2^ (IQR = 13). At diagnosis, the majority of patients (*n* = 399; 79.2%) were classified as Fédération Internationale de Gynécologie et d’Obstétrique (FIGO) stage I, representing the largest subgroup, followed by stage III with 52 patients (10.4%). A small number of patients (*n* = 3; 0.6%) were diagnosed with FIGO stage IV disease.

Tumor staging according to the Tumor, Node, Metastasis (TNM) classification revealed that most tumors were T1a (tumor invading less than half of the myometrium), accounting for 146 cases (29%), while 108 cases (24.4%) involved superficial tumors with tumor invading more than half of the myometrium. The second most common T stage was T1b, comprising 145 cases (28.8%). Higher T stages were less frequent; only three patients (0.6%) had T4 disease. Complete staging data are presented in Table 1.

With respect to nodal involvement, the majority of patients (*n* = 299; 59.3%) had no lymph node metastases (N0). Pelvic lymph node involvement (N1) was observed in 49 cases (9.7%), and paraaortic lymph node involvement (N2) in 12 patients (2.4%). In 144 cases (28.6%), no pathological nodal status was obtained due to the omission of operative nodal staging.

Distant metastases at diagnosis were absent in most patients: 461 patients (91.5%) had no evidence of metastasis (M0), whereas primary metastatic disease was present in 13 patients (2.6%) (Table 1).

**Figure 1:**
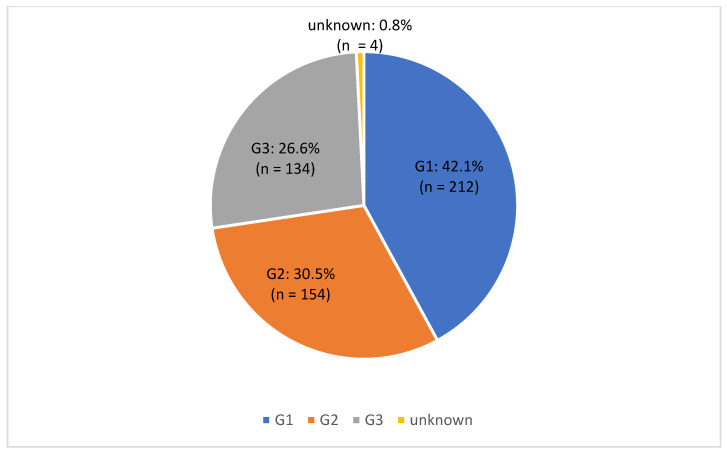
Frequencies of histological grading (G1–G3) in the study cohort.

Tumor grading revealed that G1 tumors were the most prevalent, accounting for 42.1% (*n* = 212) of cases, followed by G2 tumors in 30.5% (*n* = 154) of cases. In four patients (0.8%), data on tumor grade were missing (Figure 1).

**Figure 2:**
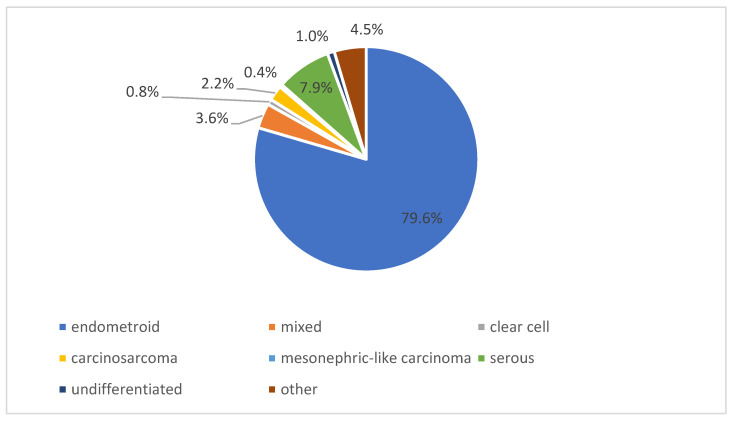
Distribution of histological subtypes of endometrial cancer in the study cohort.

Endometrioid carcinoma was the predominant histological subtype, accounting for 79.6% (*n* = 401) of cases. Serous carcinoma represented 7.9% (*n* = 40) of tumors. Other less frequent subtypes included mixed histology in 3.6% (*n* = 18), carcinosarcoma in 2.2% (*n* = 11), and undifferentiated carcinoma in 1.0% (*n* = 5) of cases. Clear cell carcinoma (0.8%, *n* = 4) and mesonephric-like carcinoma (0.4%, *n* = 2) were rare. Additional rare histological subtypes classified as other accounted for 4.5% (*n* = 23) of cases (Figure 2).

Overall, the observed distribution of histological subtypes reflects the typical spectrum of endometrial cancer encountered in tertiary care settings. The predominance of endometrioid carcinoma was consistent across all participating centers, while non-endometrioid subtypes collectively accounted for a smaller but clinically relevant proportion of cases. This heterogeneity underscores the importance of comprehensive pathological and molecular characterization in routine diagnostics.

Analysis of adjuvant therapies revealed that external beam radiotherapy (EBRT) was administered in 56 patients (11.1%), while 433 patients (85.9%) did not receive EBRT. Brachytherapy was performed in 95 patients (18.8%), whereas 392 (77.8%) did not undergo this form of adjuvant radiotherapy. Adjuvant chemotherapy was administered in 115 patients (22.8%), while 378 (75.0%) did not receive chemotherapy. Immune checkpoint inhibitors were used only in 6 patients (1.2%; Table 2).

The distribution of molecular and immunohistochemical markers was also assessed (*n* = 504). For estrogen receptor (ER) and progesterone receptor (PR), 46.6% and 44.2% of cases were positive, while 7.1% and 8.3% were negative; however, nearly half of the cases lacked data for these markers. L1 cell adhesion molecule (L1CAM) expression was positive in 1.4% and negative in 6.7% of tumors, with 91.9% missing. Lymphovascular space invasion (LVSI) was positive in 4.2% and negative in 22.2% of cases, with 73.6% lacking data. *TP53* was mutated in 9.3% and wild-type in 24.2% of cases, with two-thirds missing. *POLE* mutations were rare (0.8%), with 7.5% wild-type and 91.7% unknown.

**Figure 3:**
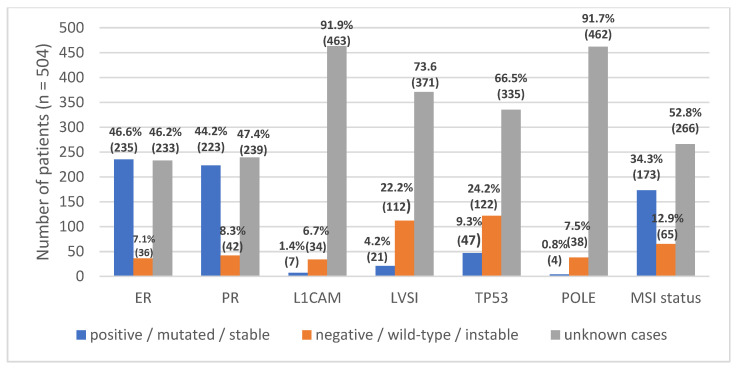
Frequencies of molecular and histological markers in endometrial cancer: estrogen receptor (ER), progesterone receptor (PR), L1 cell adhesion molecule (L1CAM), lymphovascular space invasion (LVSI), TP53, and POLE alterations and Microsatellite instability status across all cases.

Regarding microsatellite status, 34.3% of tumors were microsatellite stable, 12.9% showed MSI, and 52.8% had missing data. Overall, a substantial proportion of cases lacked complete molecular annotation across markers, indicating considerable variability in data completeness (Figure 3).

**Figure 4:**
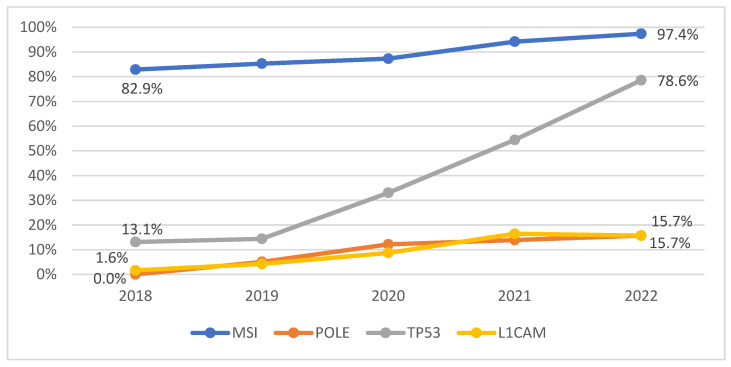
Development of annual testing rate for MSI-, *POLE-*, *TP53*- and L1CAM-Testing between 2018–2022.

There was a significant increase in the annual rates of molecular testing for MSI, *POLE*, *TP53*, and L1CAM over the five-year observation period (all *p* < 0.05). The largest relative increase was observed for TP53 testing, which rose from 13.1% in 2018 to 78.6% in 2022. In contrast, MSI testing showed a smaller rise, from an already high baseline of 82.9% to 97.4%. Notably, both *POLE* and L1CAM testing were virtually absent in 2018 (0% and 1.6%, respectively) but increased significantly to 15.7% by 2022 (*p* < 0.05; Figure 4).

## 4. Discussion

### 4.1. Principal Findings

This multicenter retrospective study demonstrates a significant rise in molecular testing—particularly for MMR and MSI—in patients with endometrial carcinoma in Germany between 2018 and 2022. The rate of MSI testing increased from 82.9% to 97.4%, reflecting a rapid adoption of updated national and international guideline recommendations [6,7,8]. Nevertheless, testing for additional The Cancer Genome Atlas (TCGA)-related markers such as *POLE*, *TP53*, L1CAM, and LVSI remained inconsistent, with more than half of the cohort lacking complete molecular annotation. Despite the growing implementation of molecular diagnostics, only 1.2% of patients received immune checkpoint inhibitors, which reflects the time gap between practice-changing study results and approval.

### 4.2. Comparison with Existing Literature

Our findings align with recent European real-world data. In the ECHO-EU-1L/2L study, only about one-third of women with advanced or recurrent endometrial cancer underwent MSI testing prior to 2021, despite clear recommendations for universal testing [9]. It should be noted that our study cohort included newly diagnosed patients, while the ECHO-EU-1L/2L included advanced or recurrent endometrial cancer. Similarly, national audits in the United Kingdom and Scandinavia have shown incomplete adherence to MMR testing guidelines, particularly outside academic centers [6]. In contrast, U.S. registry analyses reported rapid increases in testing rates following NCCN guideline updates, with >90% of cases tested after 2022 [6].

The incomplete testing of additional markers (*POLE*, *TP53*, L1CAM) observed in our study mirrors prior reports of heterogeneous implementation of the full TCGA classification [10,11]. The clinical relevance of these molecular subtypes is now well established: *TP53*-mutant tumors are associated with poorer outcomes, while *POLE*-mutated tumors show excellent survival [5]. This is further supported by Casanova et al., who confirmed significantly improved survival in *POLE*-mutated high-grade endometrioid tumors, reinforcing the necessity of consistent *POLE* testing [12]. Moreover, discordances between immunohistochemistry-based MMR assessment and MSI testing, as reported by Geurts et al., emphasize the need for standardized, quality-assured protocols [13,14].

Therapeutically, the identification of MMR deficiency or MSI-high (MSI-H) status has become essential, given recent phase III evidence. The NRG-GY018 trial demonstrated a substantial progression-free survival benefit for pembrolizumab plus platinum-based chemotherapy in patients with advanced or recurrent endometrial cancer [15]. Similarly, the RUBY trial confirmed improved outcomes with dostarlimab in the deficient mismatch repair (dMMR)/MSI-H subgroup [16,17]. These data are complemented by the recent KEYNOTE-B21 trial, which, although negative in the overall population, demonstrated a disease-free survival benefit in the dMMR subgroup, further emphasizing the clinical relevance of universal MMR testing in adjuvant settings [18]. Meta-analyses and reviews further underline that immune checkpoint inhibitors yield the greatest efficacy in this subgroup but remain underutilized in practice [10,19]. Beyond diagnostic availability, the translation of molecular findings into therapeutic decision-making remains a critical challenge. Although MMR and MSI testing rates increased substantially during the study period, the clinical consequences of these results were not always reflected in treatment patterns. This observation highlights a potential implementation gap between diagnostic knowledge and therapeutic application.

Several factors may contribute to this discrepancy, including delayed drug approvals, reimbursement constraints, limited interdisciplinary coordination, and varying levels of familiarity with emerging evidence. In addition, the rapid evolution of molecular classifications and treatment algorithms poses challenges for timely integration into established clinical workflows.

Strengthening interdisciplinary tumor boards, improving access to molecular tumor profiling, and fostering continuous guideline updates may help bridge this gap. Real-world analyses such as the present study are essential to identify barriers to implementation and to inform targeted interventions aimed at optimizing personalized treatment strategies for patients with endometrial cancer.

### 4.3. Implications for Clinical Practice

The observed rise in MMR and MSI testing marks an important step toward precision oncology in endometrial cancer. Identification of MMR deficiency informs adjuvant therapy selection, guides use of immunotherapy, and facilitates detection of Lynch syndrome [6,7,8]. However, incomplete molecular profiling limits the full integration of TCGA-based risk classification into daily clinical decision-making. To bridge this gap, routine universal MMR/MSI testing at diagnosis should be complemented by systematic assessment of *POLE* and *TP53* status, standardized testing algorithms. Recent comparative analyses demonstrate strong concordance between IHC and molecular MSI testing, though discordance—particularly in isolated *MSH6* loss—highlights the importance of standardized, quality-controlled workflows [20]. Importantly, molecular findings must be translated into actionable treatment choices. Due to the retrospective study design and incomplete availability of follow-up and outcome data across centers, correlation analyses between molecular biomarkers, tumor stage, treatment modalities, and clinical outcomes were not feasible. The low rate of immune checkpoint inhibitor use in our cohort illustrates that some centers started to administer immunotherapy in high-risk settings based on trial results.

### 4.4. Future Research

Future research should focus on prospective, nationwide implementation studies to evaluate barriers to comprehensive molecular testing and to monitor its real-world impact on survival and recurrence. Health-services and cost-effectiveness analyses are needed to support equitable access to molecular diagnostics across healthcare settings. Further biological investigations should aim to refine predictive biomarkers, including combined genomic and immune signatures such as tumor mutational burden and immune infiltration, to identify patients most likely to respond to PD-1 blockade. Recent reviews indicate that up to half of MSI-H/dMMR tumors fail to respond to checkpoint inhibitors, underscoring the need for next-generation biomarker discovery [10]. Ultimately, aligning molecular diagnostics, treatment pathways, and patient access will be key to realizing the full potential of personalized therapy in endometrial cancer.

## 5. Conclusions

MMR and MSI testing have rapidly transitioned from trial results to clinical standard in Germany between 2018 and 2022. Despite significant diagnostic progress, incomplete molecular profiling and limited use of immunotherapy highlight a persistent gap between testing and treatment implementation. Systematic integration of comprehensive molecular diagnostics will be the key to fully realizing the benefits of precision oncology in endometrial cancer.

## Figures and Tables

**Table 1:** Baseline characteristics of the study cohort (*n* = 504).

Characteristic	*n* (%) or Median (IQR)
**Age (years)**	Median 63 (14.75)
**BMI (kg/m^2^)**	Median 30 (13)
**T stage**	T1a with myometrial invasion: 146 (29.0%) T1a without myometrial invasion: 108 (21.4%) T1b: 145 (28.8%) T2: 49 (9.7%) T3a: 23 (4.6%) T3b: 29 (5.8%) T4: 3 (0.6%) unknown: 1 (0.2%)
**N stage**	N0: 299 (59.3%) N1: 49 (9.7%) N2: 12 (2.4%) unknown: 144 (28.6%)
**M stage**	M0: 461 (91.5%) M1: 13 (2.6%) unknown: 30 (6.0%)

**Table 2:** Adjuvant therapies administered in the study cohort.

Adjuvant Therapy	*n* (%)
Adjuvant radiotherapy, external beam radiotherapy (EBRT)	
yes	56 (11.1%)
no	433 (85.9%)
unknown	15 (3.0%)
Adjuvant radiotherapy, brachytherapy	
yes	95 (18.8%)
no	392 (77.8%)
unknown	17 (3.4%)
Adjuvant chemotherapy	
yes	115 (22.8%)
no	378 (75.0%)
unknown	11 (2.2%)
Adjuvant immune checkpoint inhibitor	
yes	6 (1.2%)
no	489 (97.0%)
unknown	9 (1.8%)
Adjuvant endocrine therapy	
yes	3 (0.6%)
no	492 (97.6%)
unknown	9 (1.8%)

## Data Availability

The data presented in this study are not publicly available due to privacy and ethical restrictions. Anonymized data may be available from the corresponding author upon reasonable request, subject to institutional and data protection regulations.

## Abbreviations

The following abbreviations are used in this manuscript:

AWMF	Association of the Scientific Medical Societies in Germany
BMI	Body Mass Index
dMMR	Deficient mismatch repair
EBRT	External beam radiotherapy
EC	Endometrial cancer
ER	Estrogen receptor
FIGO	Fédération Internationale de Gynécologie et d’Obstétrique
IHC	Immunohistochemistry
IQR	Interquartile range
L1CAM	L1 cell adhesion molecule
LVSI	Lymphovascular space invasion
MMR	Mismatch repair
MSI	Microsatellite instability
MSI-H	Microsatellite instability high
PCR	Polymerase chain reaction
POLE	Polymerase epsilon
PR	Progesterone receptor
TCGA	The Cancer Genome Atlas
TNM	Tumor, Node, Metastasis
TP53	Tumor protein p53

## References

[1] Siegel R.L., Miller K.D., Jemal A. (2020). Cancer statistics, 2020. CA Cancer J. Clin..

[2] Robert Koch Institute (2023). Cancer in Germany 2019/2020.

[3] Casey L., Singh N. (2021). POLE, MMR, and MSI testing in endometrial cancer: Proceedings of the ISGyP companion society session at the USCAP 2020 annual meeting. Int. J. Gynecol. Pathol..

[4] Emons G., Steiner E., Vordermark D., Uleer C., Paradies K., Tempfer C., Aretz S., Cremer W., Hanf V., Mallmann P. (2018). S3 Guideline for Endometrial Cancer (Long Version). Guideline of the German Society of Gynecology and Obstetrics (DGGG), German Cancer Society (DKG) and Association of the Scientific Medical Societies in Germany (AWMF).

[5] Riedinger C.J., Esnakula A., Haight P.J., Suarez A.A., Chen W., Gillespie J., Villacres A., Chassen A., Cohn D.E., Goodfellow P.J. (2024). Characterization of mismatch repair/microsatellite instability–discordant endometrial cancers. Cancer.

[6] Concin N., Matias-Guiu X., Vergote I., Cibula D., Mirza M.R., Marnitz S., Ledermann J., Bosse T., Chargari C., Fagotti A. (2021). ESGO/ESTRO/ESP guidelines for the management of patients with endometrial carcinoma. Int. J. Gynecol. Cancer.

[7] Emons G., Steiner E., Vordermark D., Uleer C., Paradies K., Tempfer C., Aretz S., Cremer W., Hanf V., Mallmann P. (2023). Endometrial cancer. Guideline of the DGGG, DKG and DKH (S3-Level, AWMF Registry No. 032/034-OL, September 2022)—Part 2. Geburtshilfe Frauenheilkd..

[8] Emons G., Steiner E., Vordermark D., Uleer C., Paradies K., Tempfer C., Aretz S., Cremer W., Hanf V., Mallmann P. (2023). Endometrial Cancer. Guideline of the DGGG, DKG and DKH (S3-Level, AWMF Registry Number 032/034-OL, September 2022). Part 1 with Recommendations on the Epidemiology, Screening, Diagnosis and Hereditary Factors of Endometrial Cancer, Geriatric Assessment and Supply Structures. Geburtshilfe Frauenheilkd..

[9] Kelkar S.S., Prabhu V.S., Zhang J., Ogando Y.M., Roney K., Verma R.P., Miles N., Marth C. (2024). Real-world prevalence of microsatellite instability testing and related status in women with advanced endometrial cancer in Europe. Arch. Gynecol. Obstet..

[10] Marchetti M., Ferrari J., Vezzaro T., Masatti L., Tasca G., Maggino T., Tozzi R., Saccardi C., Noventa M., Spagnol G. (2024). The role of immunotherapy in MMR-deficient endometrial carcinoma: State of the art and future perspectives. J. Clin. Med..

[11] Mendiola M., Heredia-Soto V., Ruz-Caracuel I., Baillo A., Ramon-Patino J.L., Escudero F.J., Miguel M., Pelaez-Garcia A., Hernandez A., Feliu K. (2023). Comparison of methods for testing mismatch repair status in endometrial cancer. Int. J. Mol. Sci..

[12] Casanova J., Duarte G.S., da Costa A.G., Catarino A., Nave M., Antunes T., Serra S.S., Dias S.S., Abu-Rustum N., Lima J. (2024). Prognosis of polymerase epsilon (POLE) mutation in high-grade endometrioid endometrial cancer: A systematic review and meta-analysis. Gynecol. Oncol..

[13] Geurts B.S., Zeverijn L.J., van Berge Henegouwen J.M., van der Wijngaart H., Hoes L.R., de Wit G.F., Spiekman I.A.C., Battaglia T.W., van Beek D.M., Roepman P. (2024). Characterization of discordance between mismatch repair deficiency and microsatellite instability testing may prevent inappropriate treatment with immunotherapy. J. Pathol..

[14] Berg H.F., Engerud H., Myrvold M., Lien H.E., Hjelmeland M.E., Halle M.K., Woie K., Hoivik E.A., Haldorsen I.S., Vintermyr O. (2023). Mismatch repair markers in preoperative and operative endometrial cancer samples: Expression concordance and prognostic value. Br. J. Cancer.

[15] Eskander R.N., Sill M.W., Beffa L., Moore R.G., Hope J.M., Musa F.B., Mannel R., Shahin M.S., Shahin G.H., Girda E. (2023). Pembrolizumab plus chemotherapy in advanced endometrial cancer. N. Engl. J. Med..

[16] Mirza M.R., Chase D.M., Slomovitz B.M., dePont Christensen R., Novák Z., Black D., Gilbert L., Sharma S., Valabrega G., Landrum L.M. (2023). Dostarlimab for primary advanced or recurrent endometrial cancer. N. Engl. J. Med..

[17] Powell M.A., Bjørge L., Willmott L., Novák Z., Black D., Gilbert L., Sharma S., Valabrega G., Landrum L.M., Gropp-Meier M. (2024). Overall survival in patients with endometrial cancer treated with dostarlimab plus carboplatin–paclitaxel in the randomized ENGOT-EN6/GOG-3031/RUBY trial. Ann. Oncol..

[18] Van Gorp T., Cibula D., Lv W., Backes F., Ortaç F., Hasegawa K., Lindemann K., Savarese A., Laenen A., Kim Y.M. (2024). ENGOT-en11/GOG-3053/KEYNOTE-B21: A randomized, double-blind, phase III study of pembrolizumab or placebo plus adjuvant chemotherapy with or without radiotherapy in patients with newly diagnosed high-risk endometrial cancer. Ann. Oncol..

[19] Zhu Y., Liu K., Zhu H. (2024). Immune checkpoint inhibitor combinations for patients with advanced endometrial cancer: A network meta-analysis and cost-utility analysis. Int. J. Gynecol. Cancer.

[20] Sowter P., Gallon R., Hayes C., Phelps R., Borthwick G., Prior S., Combe J., Buist H., Pearlman R., Hampel H. (2024). Detection of mismatch repair deficiency in endometrial cancer: Assessment of IHC, fragment length analysis, and amplicon sequencing-based MSI testing. Cancers.

